# A missense mutation in *ISPD* contributes to maintain muscle fiber stability

**DOI:** 10.1016/j.psj.2022.102143

**Published:** 2022-08-31

**Authors:** Lijin Guo, Siyu Zhang, Yibin Xu, Yulin Huang, Wei Luo, Qi Wen, Guanxuan Liu, Weiling Huang, Haiping Xu, Biao Chen, Qinghua Nie

**Affiliations:** ∗ Guangdong Provincial Key Lab of Agro-Animal Genomics and Molecular Breeding, and Key Laboratory of Chicken Genetics, Breeding and Reproduction, Ministry of Agriculture, College of Animal Science, South China Agricultural University, Guangzhou, Guangdong, China; † College of Animal Science & Technology, Jiangxi Agricultural University, Nanchang, Jiangxi, China; ‡ State Key Laboratory of Livestock and Poultry Breeding, Guangdong Key Laboratory of Animal Breeding and Nutrition, Guangdong Public Laboratory of Animal Breeding and Nutrition, Institute of Animal Science, Guangdong Academy of Agricultural Sciences, Guangzhou, China

**Keywords:** *ISPD*, missense mutation, myogenesis, atrophy antagonism, myofiber stability

## Abstract

**Background:**

Livestock and poultry skeletal muscle development was regulated by a variety of genetic factors playing an essential role in this process. In our de novo sequencing, a missense mutation in *ISPD* exon 2 showed a selected signature for well-developed muscles. However, the relationship between this mutation and muscle phenotypes remains unclear.

**Results:**

Based on the genotype bias of this missense mutation in gamecock chickens, we compared the cross-section of muscle fibers among the individuals with different genotypes with the results showing a genotype preference of this missense mutation in the chickens with hypertrophic muscle fibers. Comparing TT- and CC-genotype *ISPD* showed that TT- genotype was associated with heavier skeletal muscle and a better effect on myogenesis and myofiber stability. At cellular level, *ISPD* was identified to inhibit myoblast proliferation, differentiation and antagonize of muscular atrophy.

**Conclusion:**

It can be concluded that the missense mutation of Arg84Lys in *ISPD* was associated with well-developed muscles and demonstrated to improve chicken muscles’ development.

## INTRODUCTION

The growth and development of skeletal muscle has been a hot topic in agricultural sciences, and many key factors involved in this process have been identified (Dodson et al., 2010; Chen et al., 2013; Grosicki et al., 2018; Wang et al., 2019; Yang et al., 2021). In genetics, animal genome diversity is an important consideration for animal breeding (Dunn and Ryan, 2015; Li et al., 2017; Bhati et al., 2020; Cai et al., 2020). Genomic polymorphisms are strongly associated with growth traits in livestock animals, especially muscle growth phenotypes. Typically, the loss of function mutations in of myostain (***MSTN***) leads to increasing body weight and muscle mass in varied species (Mcpherron and Lee, 1997; Mcpherron et al., 1997; Boman et al., 2010; Ren et al., 2020, Kim et al., 2020, Lee et al., 2021). The sex-linked dwarf chicken carry *GHR* mutations represents a valuable animal model characterized by both a small size and reduced muscle mass (Agarwal et al., 1994). These characteristics are important phenotypes in the livestock industry to improve carcass traits, suggesting the essential effect of genomic polymorphisms on livestock muscular phenotypes.

Compared with commercial broilers, gamecocks were characterized with recovery capability and developed muscle to adapt frequent fighting behaviors. In our previous study (Luo et al., 2020), Isoprenoid synthase domain containing (***ISPD***) was speculated as a candidate for the difference between gamecocks and commercial broilers. N-terminal cytidyltransferase domain was showed in ISPD crystallization and acts independently from protein O-mannosyltransferase (**POMT**) activity (Riemersma et al., 2015), indicating that ISPD is a cytidyltransferase and required for dystroglycan O-mannosylation. Loss of function mutations in *ISPD* was reported to be associated with congenital muscular dystrophy by disrupting dystroglycan O-mannosylation (Vuillaumier-Barrot et al., 2012; Willer et al., 2012), causing the abolishment of the initial step in laminin-binding glycan synthesis. Distinguishing from the mevalonate pathway commonly used in most eukaryotes and archaebacteria, ISPD was implicated in isopentenyl pyrophosphate formation, which serve as precursors for isoprenoid modifications of protein (Rohdich et al., 2000; Grawert et al., 2011). Besides, ISPD synthesized CDP-ribitol and both recombinant FKTN (fukutin) and FKRP (fukutin-related protein) transfer a ribitol phosphate group from CDP-ribitol to alpha-dystroglycan, and this process was specific in muscle (Gerin et al., 2016), suggesting that ISPD was highly linked with muscular phenotypes. However, the effect of *ISPD* on muscular phenotypes in chicken has not been reported.

In this study, we explored the polymorphism preference of *ISPD* in different chickens with different muscular phenotypes, and compared the effect of different *ISPD* genotypes on muscle fiber maintenance at cellular level.

## MATERIALS AND METHODS

### Ethics Approval and Animal Experiments

All animal experiments in this research were approved by the Animal Care Committee of South China Agricultural University (Approval number: SCAU#C029). 723 Mahuang Chickens, 80 days old, were sacrificed by exsanguination of the neck, blood was collected for subsequent DNA extraction. Their growth traits were measured, including body weight, slaughtering weight, half-eviscerated weight, eviscerated weight, breast muscle weight, wing weight, foot weight, head weight, heart weight, liver weight, stomach weight, abdominal fat weight, shank length, shank circumference and body length.

### Cell Culture

Chicken primary myoblast was isolated from the leg muscle of 11th embryonic age Mahuang chicks with wild type *ISPD*. 2.5% Trypsin (Gibco, CA) was used in muscle tissues dissociation. DMEM medium (Gibco, CA) with 20% fetal bovine serum (Gibco, CA) and 1% streptomycin/penicillin (Invitrogen, CA) was used to culture myoblast. The myoblast was cultured in an incubator (ThermoFisher, NY) at 37°C with 5% CO_2_.

### RNA Extraction and cDNA Synthesis

The total RNA from cells and tissues was extracted by using a HiPure Total RNA Midi Kit (Magen, Guangzhou China) following its instruction. The extracted RNA was used in subsequent cDNA synthesis. MonScript RTIII All-in-One Mix with dsDNase (Monad, Wuhan, China) was used in cDNA first strand synthesis according to its manufacture instruction.

### Quantitative Real-time PCR (qRT-PCR)

qRT-PCR was used to quantify relative mRNA expression. Quantitative real-time PCR was carried out in an ABI QuantStudio 5 instrument (ThermoFisher, NY) using ChamQ Universal SYBR qPCR Master Mix (Vazyme, Nanjing, China). GAPDH was used as a reference gene in this study. The primers were designed in Primer-Blast (accession link: https://www.ncbi.nlm.nih.gov/tools/primer-blast). The primers were synthesized by Tsingke Biological Technology company (Guangzhou China) and they were listed in Supplementary Table 1.

### Hematoxylin and Eosin (H&E) Staining

After the neck dislocation treatment, 7 Dongtao chickens, 9 Bearded chickens and 27 Mahuang chickens were sacrificed, their leg muscles (gastrocnemius) and breast muscle were collected respectively and immersed in 4% paraformaldehyde (Solarbio, Beijing, China). After 24-h fixation, the muscle tissues were embedded in paraffin. Subsequently, histotome instrument (Leica, Wetzlar, Germany) was used to prepare the muscular transverse sections. The sections were stained with hematoxylin and eosin. Finally, the random screens of the sections were captured in a microscope (Nikon, Tokyo, Japan).

### Plasmid Construction

The CDS sequence of *ISPD* transcript (ENSGALT00000093602.1) was synthesized by Tsingke Biological Technology Company (Guangzhou China). The synthesized sequence was cloned into the XhoI site and XbaI site of pcDNA3.1 vector (Pormega, CA), and named as ISPD-CC. The nucleotide of position 270 in this transcript was changed to A from G. This mutated sequence was also synthesized and cloned into pcDNA3.1 vector, and named as ISPD-TT. Plasmid extraction was performed by using a HiPure Plasmid EF Mini Kit (Magen, Guangzhou China) following its manufacture instruction.

### Cell Transfection

For cell transfection, Lipofectamine 3000 Reagent (Invitrogen, CA) was used in plasmid transfection. The plasmid transfection dose was: 2.5 μg/well for 6-well plate, 1 μg/well for 12-well plate and 0.25 μg/well for 48-well plate.

### ISPD Cloning and Genotyping

The sequence in *ISPD* containing with rs15077795 site was cloned by using 2 × MasterTaq Mix (CWBIO, Taizhou, China). The PCR product was sent to Tsingke Biological Technology Company (Guangzhou, China) for SANGER sequencing. The primers used in PCR amplification were designed in Primer-Blast (accession link: https://www.ncbi.nlm.nih.gov/tools/primer-blast) and their information was listed in the Supplementary Table 1. The SANGER sequencing results were visualized in DNAstar software, the genotypes of alleles were identified according to visualized peak.

### 5-ethynyl-2’-deoxyuridine (EdU) Assay

EdU was used to visualize cell proliferation activity in this study. The Cell-Light EdU Apollo 567 In Vitro Kit was purchased from RIBOBIO company (Guangzhou, China). The specific steps of EdU assay were carried out by following the kit's instruction. The staining cells’ random screens were captured in a DMi8 microscope (Leica, Wetzlar, Germany).

### Flow Cytometry Assay

After 48-h treatment, the cells were digested by using 0.25%-EDTA trypsin (Gibco, CA) and they were centrifuged at 3,000 × g at 4°C for 5 min. Next, the cell pellet was washed and resuspended twice in ice-cold PBS (Gibco, CA). After another centrifuge, the cells were resuspended by in ice-cold 70% ethanol (Damao, Tianjin, China) and stored at −20°C for 24 h. The fixed cells were sent Guangzhou KYDbio Technology (Guangzhou, China) for flow cytometry analysis. The fitting analysis of cell cycle results were performed in ModFit LT software.

### Cell Count Kit-8 (CCK-8) Assay

CCK-8 was used to measured cell viability by following its instruction. Briefly, at different time points, 25 μL CCK-8 reagent was added into the each well of a 48-well plate. After 60-min incubation, the light absorption value was detected in a microplate reader (BioTek, VT).

### Immunofluorescence Assay

After 6-h transfection, fetal bovine serum concentration of myoblast medium was changed from 20% to 5% for next 36-h differentiation induction. The cells were fixed by using 4% paraformaldehyde for 20 min and washed 3 times with PBS for 5 min. 0.1% Triton X-100 (Solarbio, Beijing, China) was used to permeabilize cell membrane for 5 min. Goat serum (Solarbio, Beijing, China) was used in blocking for 30 min. After that, the cells were incubated with anti-MyHC (1:50, DHSB, IA) overnight at 4°C and incubate with DyeLight-549 anti-mouse IgG secondary antibody (Bioworld, MN) for 1 h. DAPI (Solarbio Beijing, China) was used to nucleus staining for 5 min. Finally, stained cells were captured in a DMi8 microscope (Leica, Wetzlar, Germany).

### Western Blotting

Total protein was released from cells through lytic effect by using ice-cold radioimmunoprecipitation (**RIPA**) lysis buffer (Beyotime, Shanghai, China) with 0.5 mM/mL phenylmethylsulfonyl fluoride (Solarbio, Beijing, China). Lysate was centrifuged at 13,000 × g at 4°C for 15 min. The protein supernatant was transferred in a new tube and was incubated in 98°C with Protein Buffer (TransGen Biotech, Beijing, China) for 5 min. Same amount of protein was separated in a 12% SDS-PAGE, and transferred to PVDF membranes (BioRad, CA). QuickBlock Blocking Buffer (Byotime, Shanghai, China) was used to membranes incubation for 30 min at room temperature. Blocked membranes were incubated with antibodies against MyoD (1:500, NovusBio, CO), MyHC (1:200, DHSB, IA), MurF-1 (1:500, Proteintech, IL), Atrogin-1 (1:1000, Abcam, Cambridge, UK) and GAPDH (1:2000, Bioworld, MN) overnight at 4°C. Next, anti-mouse or anti-rabbit IgG secondary antibodies with HRP label were used to incubate the membranes for 1 h at room temperature. High sensitivity ECL chemiluminescence detection kit (Vazyme, Nanjing, China) was used in chromogenic reaction by following the instruction. Chromogenic bands were captured in an Odyssey instrument (Li-cor, CA). The gray value of bands was measured in ImageJ software.

### Statistics and Analysis

A total of 24 traits per chicken were used in association analysis with *ISPD* SNPs. For some individuals, the traits record was not complete, therefore, the individual number of traits for every SNP may be different. SAS 9.4 software was used to performed the association analysis between SNPs and traits. The genetic effect of alleles’ genotypes was analyzed following the mixed procedure model:

Y=μ+F+M+S+G+e

where *Y* represents the traits’ phenotypic values; *μ* represents total population mean; *F*, the effect of father; *M*, the effect of mother; *S*, the effect of gender; *G*, the effect of genotype; *e*, random residuals.

All the results were shown as mean ± SEM, and the differences between groups were calculated by Student's t-test or one-way ANOVA analysis.

## RESULTS

### Genotype Preference of *ISPD* in Hypertrophic Muscle Fibers

**Figure 1:**
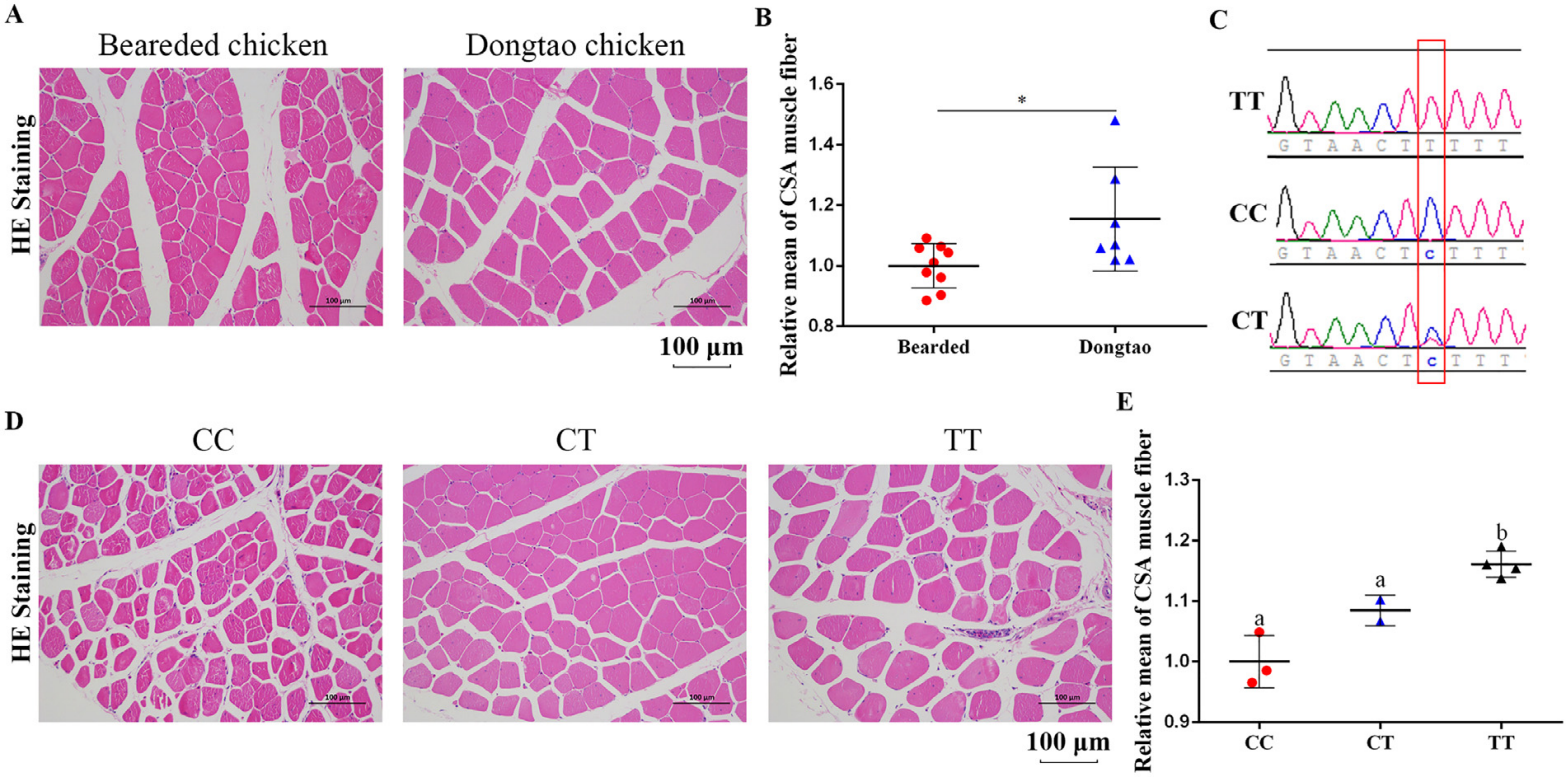
The genotypes preference of ISPD among the chickens. (A) The H&E staining of leg muscle isolated from 14-wk-old Beareded chickens and 14-wk-old Beareded chickens. (B) Relative mean of cross-section area muscle fiber. (C) Three genotype peak diagrams of Arg84Lys missense mutation. (D) The H&E staining of leg muscle isolated from 14-wk-old Beareded chickens with different genotypes. (E) Relative mean of cross-section area muscle fiber in 14-wk-old Beareded chickens.

**Figure 2:**
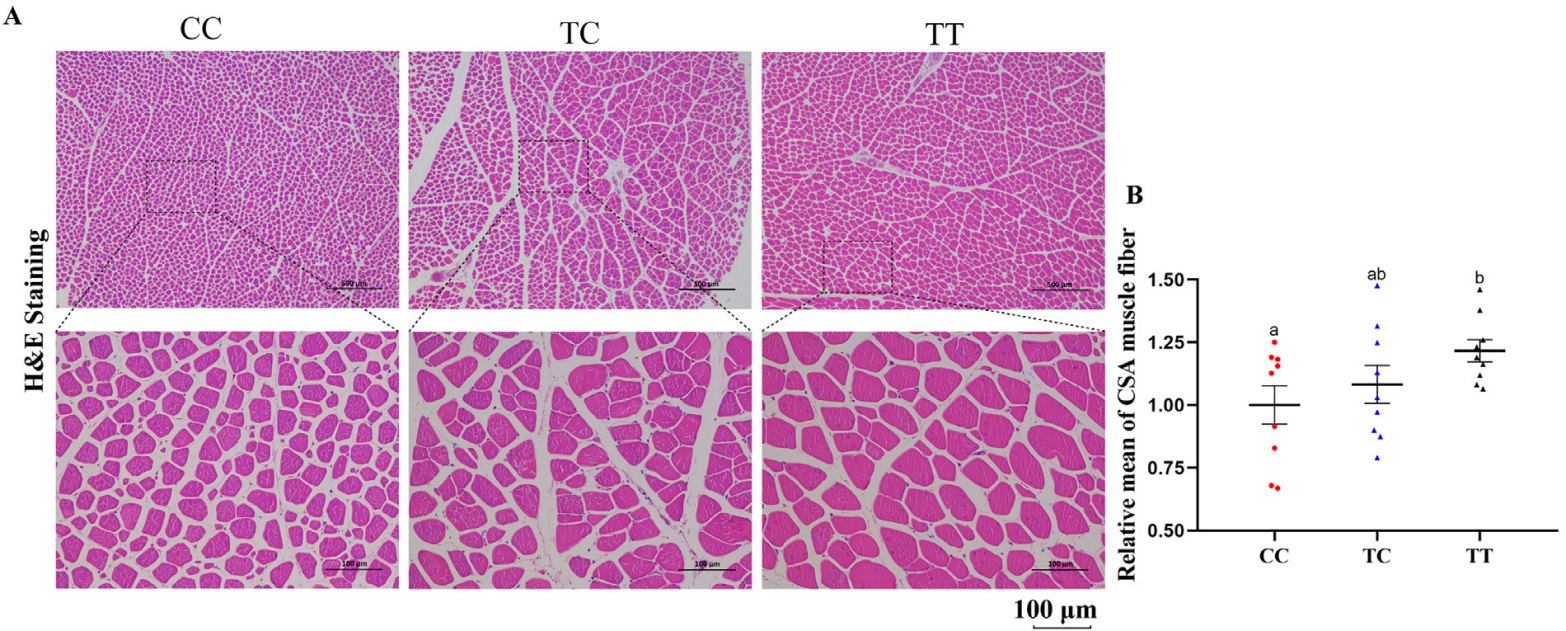
The comparison of Mahuang chicken breast muscle fibers under different genotypes. (A) The H&E staining of breast muscle isolated from 80-day-old Mahuang chickens with different genotypes. (B) Relative mean of cross-section area muscle fiber in 80-day-old Mahuang chickens.

It is well known that gamecock chickens have hypertrophic muscle fibers to adapt to more fighting behaviours. Based on the previous de novo sequencing, *ISPD* was selected as a candidate gene which may be associated with muscular signatures of gamecock chickens and it was found that the missense mutation of Arg84Lys in *ISPD* was widespread, indicating it is responsible for the enhanced muscle mass. Here, in order to explore whether the mutation was widely associated with chicken muscular traits, we used Dongtao chickens (n = 7) as the broiler represents with hypertrophic muscle fibers, while Bearded chickens (n = 9) were considered as the broiler represents with normal muscle fibers. It could be found that the average cross-sectional area of leg muscle fibers in Dongtao chickens was larger than that in Bearded one (Figure 1A, B). Based on this mutated site, the 16 samples were genotyped. Interestingly, the allele of the missense mutation we described above in Dongtao chicken were all TT, while the allele in Bearded chickens were CC (n = 3), CT (n = 2), or TT (n = 4) (Figure 1C). Subsequently, the leg muscle cross-sectional area (CSA) of the 9 Bearded chickens with different genotypes was measured (Figure 1D, E). The results showed a higher muscle fiber cross-sectional area in the individuals with CT and TT genotype, especially in TT. Similarly, it was also found that the breast muscle CSA of Mahuang chicken with TT genotype (n = 9) was significantly larger than that of CC genotype (n = 9) (Figure 2A, B), suggesting that the missense mutation of Arg84Lys may be the reason of muscle fibers hypertrophy.

### The Identification of *ISPD* Polymorphisms

Considering the potential genotype bias of *ISPD* among the individuals with different sized muscle fibers, the relationship between *ISPD* genotypes and skeletal muscle phenotypes need to be further explored. Here, the same primers used above were used again for the chicken population (n = 723) genotyping. According to the PCR SANGER sequencing results, 6 single nucleotide polymorphisms (SNPs) were obtained. As shown in Table 1, rs15077795 (C→T, missense mutation of Arg84Lys) was ubiquitous in this population. In addition, another polymorphism (rs736807298, T→C) in the exon2 was observed and it was a synonymous mutation which could not mediate amino acid change. The other 4 SNPs were intron variants, including rs741523477 (T→C), rs313358571 (T→C), rs318003785 (C→A) and rs314242692 (G→A).

**Table 1:** SNPs in *ISPD*.

SNPs rs ID	Position in genome	Allele	Mutated type
rs736807298	NC_006089.5: 28382103	T→C	Synonymous variant
rs15077795	NC_006089.5: 28382203	C→T	Missense variant
rs741523477	NC_006089.5: 28382341	T→C	Intron variant
rs313358571	NC_006089.5: 28382345	T→C	Intron variant
rs318003785	NC_006089.5: 28382438	C→A	Intron variant
rs314242692	NC_006089.5: 28382512	G→A	Intron variant

Some of SANGER sequencing results could not show all genotypes for all SNPs due to chaotic background caused by potential indel mutations. Therefore, the individual number for different alleles may be varied. Among the 6 alleles, the P-value of Hardy-Weinberg equilibrium of rs736807298 and rs741523477 was less than 0.05. The other 4 alleles, their distribution of genotypic and allele frequencies was in Hardy-Weinberg equilibrium in this population (Table 2). For the allele of rs15077795, the *P*-value of HWE was 0.9656, the allelic frequency of C was 60.17%, while the allelic frequency of T was 39.83%, suggesting that the allele could be used in the next association analysis.

**Table 2:** The alleles formation of SNPs in *ISPD*.

SNPs rs ID	Genotypes of Alleles			Allelic frequency		HWE
rs736807298	TT (n = 627)	TC (n = 75)	CC (n = 20)	T = 0.9204	C = 0.0796	<0.0001
rs15077795	CC (n = 262)	CT (n = 346)	TT (n = 115)	C = 0.6017	T = 0.3983	0.9656
rs741523477	TT (n = 628)	TC (n = 85)	CC (n = 10)	T = 0.9274	C = 0.0726	0.0006
rs313358571	TT (n = 264)	TC (n = 344)	CC (n = 115)	T = 0.6030	C = 0.3970	0.8676
rs318003785	CC (n = 261)	CA (n = 348)	AA (n = 114)	C = 0.6017	A = 0.3983	0.9108
rs314242692	GG (n = 468)	GA (n = 139)	AA (n = 16)	G = 0.8628	A = 0.1372	0.1489

HWE, P-value of Hardy-Weinberg equilibrium.

### The Association of rs15077795 with Chicken Growth Phenotypes

Here, the association analysis between the allele genotypes and growth phenotypes was performed. Because some individuals had no complete phenotype data, so the individual number for every trait may be different. As shown in Table 3, the allele of rs15077795 was associated with multiple growth phenotypes, including body weight, slaughtering weight, half-eviscerated weight, eviscerated weight, breast weight, leg weight, wing weight, foot weight, heart weight, liver weight, abdominal fat weight, shank length, and body length. It could be clearly observed that TT genotype individuals exhibited higher phenotypes in of skeletal muscle tissues, especially in leg tissue, indicating that the TT genotype had the potential advantage on muscle fibers hypertrophy and skeletal muscle development.

**Table 3:** The association of rs15077795 with chicken growth phenotypes.

Traits	P-value	Phenotypes		
		CC	CT	TT
BW (g)	<0.0001	1642.88 ± 14.10 (n = 262) a	1648.18 ± 11.79 (n = 346) b	1692.7 ± 20.47 (n = 115) c
SW (g)	<0.0001	1447.50 ± 12.35 (n = 261) a	1451.19 ± 10.34 (n = 344) a	1490.41 ± 18.21 (n = 114) b
SR (%)	0.2984	88.13 ± 0.11 (n = 261)	88.15 ± 0.10 (n = 344)	88.17 ± 0.18 (n = 114)
HEW (g)	<0.0001	1302.88 ± 11.83 (n = 262) a	1311.26 ± 9.47 (n = 345) b	1347.86 ± 16.65 (n = 115) c
HEWR (%)	0.2715	89.95 ± 0.32 (n = 261)	90.37 ± 0.09 (n = 343)	90.02 ± 0.30 (n = 114)
EW (g)	<0.0001	1082.98 ± 9.30 (n = 262) a	1084.13 ± 7.89 (n = 345) a	1112.36 ± 13.78 (n = 115) b
EWR (%)	0.4341	74.765 ± 0.14 (n = 261) ab	74.72 ± 0.11 (n = 343) a	74.30 ± 0.26 (n = 114) b
BMW (g)	0.0384	84.42 ± 0.81 (n = 262) a	83.89 ± 0.65 (n = 346) a	85.40 ± 1.00 (n = 115) b
BMWR (%)	0.3025	15.63 ± 0.10 (n = 262)	15.52 ± 0.08 (n = 345)	15.44 ± 0.13 (n = 115)
LW (g)	<0.0001	193.71 ± 1.93 (n = 262) a	195.23 ± 1.64 (n = 346) a	200.92 ± 2.79 (n = 115) b
LWR (%)	0.3626	35.75 ± 0.15 (n = 262)	35.93 ± 0.11 (n = 345)	36.10 ± 0.16 (n = 115)
BLR (%)	0.532	44.04 ± 0.40 (n = 262)	44.21 ± 0.91 (n = 346)	42.91 ± 0.46 (n = 115)
WW (g)	<0.0001	64.42 ± 0.57 (n = 262) a	64.19 ± 0.47 (n = 346) a	65.28 ± 0.95 (n = 115) b
WR (%)	0.0201	11.91 ± 0.04 (n = 262) a	11.85 ± 0.03 (n = 345) ab	11.74 ± 0.08 (n = 115) b
FW (g)	<0.0001	71.37 ± 0.90 (n = 262) a	70.92 ± 0.75 (n = 346) a	73.15 ± 1.41 (n = 115) b
HW1 (g)	0.4916	48.86 ± 0.62 (n = 262) a	48.42 ± 0.52(n = 346) a	49.47 ± 0.86 (n = 115) b
HW2 (g)	<0.0001	7.36 ± 0.11 (n = 261) a	7.57 ± 0.12(n = 346) ab	7.63 ± 0.18 (n = 115) b
LW2 (g)	0.0004	32.31 ± 0.36 (n = 261) a	32.94 ± 0.35(n = 345) ab	33.29 ± 0.55 (n = 115) b
SW2 (g)	0.1284	31.41 ± 0.39 (n = 262)	30.98 ± 0.32(n = 346)	31.84 ± 0.52 (n = 115)
AFW (g)	<0.0001	36.32 ± 0.98 (n = 262) a	38.21 ± 0.82(n = 346) b	42.17 ± 1.56 (n = 115) c
AFR (%)	<0.0001	2.22 ± 0.06(n = 262) a	2.31 ± 0.05 (n = 346) a	2.50 ± 0.09 (n = 115) b
SL (mm)	<0.0001	69.61 ± 0.44 (n = 262) a	70.21 ± 0.36(n = 345) b	71.43 ± 0.66 (n = 114) c
SC (mm)	0.0143	11.28 ± 0.07 (n = 262) a	11.11 ± 0.06(n = 345) b	11.20 ± 0.10 (n = 114) ab
BL (cm)	<0.0001	19.48 ± 0.07 (n = 262) a	19.48 ± 0.06(n = 345) a	19.71 ± 0.11 (n = 114) b

AFW, abdominal fat weight; AFR, abdominal fat rate; BL, body length; BLR, rate of breast with leg weight; BMW, breast muscle weight; BMWR, breast muscle weight rate; BW, body weight; EW, eviscerated weight; EWR, eviscerated weight rate; FW, foot weight; HEW, half-eviscerated weight; HEWR, half-eviscerated weight rate; HW1, head weight; HW2, heart weight; LW2, liver weight; SW1, slaughtering weight; SR, slaughtering rate; SW2, stomach weight; SL, shank length; SC, shank circumference; WW, wing weight; WR, weight rate.

The phenotype data was shown as Mean±SEM. Different letters indicate differences between groups.

Association analysis with phenotypes was also carried out for rs313358571 (Supplementary Table 2). Although some *P*-values did not reach the standard of less than 0.05, these mutations still got rise to various phenotypes, including breast muscle weight and leg weight, suggesting that the SNP may also be responsible for the skeletal muscle development.

### The Missense Mutation of *ISPD* Relieves Myoblast Proliferation Inhibition

**Figure 3:**
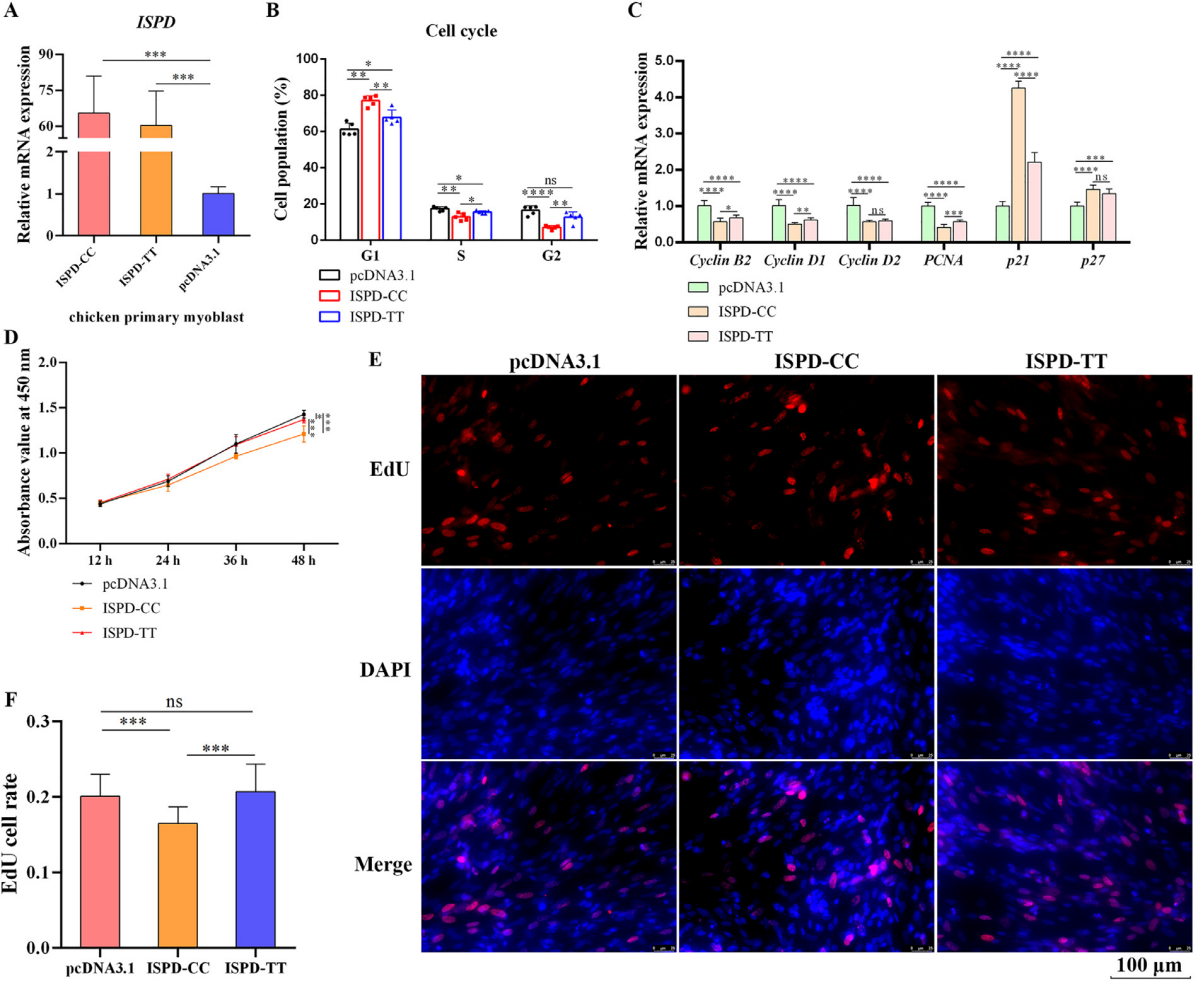
The missense mutation of Arg84Lys in *ISPD* diminishes myoblast proliferation inhibition. (A) The transfection efficiency of ISPD-CC and ISPD-TT. (B) The cell cycle of myoblast was analyzed by flow cytometry. (C) The relative mRNA levels of cell cycle-related genes. (D) Cell viability of myoblast was measured by CCK-8 assay. (E) Myoblast proliferation was detected by EdU analysis. (F) EdU-staining cell statistics.

Matching with our previous expectation, the rs15077795 genotype bias of *ISPD* was indeed associated with muscle fibers hypertrophy and skeletal muscle development. To verify how the specific allele genotype (TT) is involved with muscle development, we amplified *ISPD* CDS sequences and constructed the wild type *ISPD* overexpression plasmid (ISPD-CC) and mutated type *ISPD* overexpression plasmid (ISPD-TT). They were transfected into myoblasts and the transfection efficiency was shown in Figure 3A, which shows the successful transfections. After 48-h transfection, flow cytometry analysis was carried out. The ectopic expression of *ISPD* significantly impeded myoblast cell cycle from G1/G0 phase to S phase and G2/M phase. Interestingly, ISPD-TT dramatically released this inhibitory effect on cell cycle progress (Figure 3B). The mRNA levels of some cell cycle related genes were quantified by qRT-PCR and the result showed that *ISPD* could downregulated cell cycle-promoting genes expression while upregulated cell cycle-inhibition genes expression. Similarly, ISPD-TT also restore this effect (Figure 3C). Based on this situation, we infer that *ISPD* may suppress myoblast proliferation and the effect is weaken when a missense mutation of Arg84Lys occurs in *ISPD*. To verify this speculation, CCK-8 assay and EdU assay were performed. As shown in Figure 3D, both ISPD-CC and ISPD-TT significantly inhibited myoblasts viability. However, relative to ISPD-CC, the myoblasts cell viability under ISPD-TT ectopic expression were higher, indicating ISPD-TT did release myoblast activities inhibition. In addition, ISPD-CC obviously decreased EdU-staining cell rate and ISPD-TT release the EdU-staining cell rate inhibition (Figure 3E, F), demonstrating the missense mutation of Arg84Lys in *ISPD* could relieve myoblast proliferation inhibition.

### The Missense Mutation of Arg84Lys in *ISPD* Restores Myoblast Differentiation

**Figure 4:**
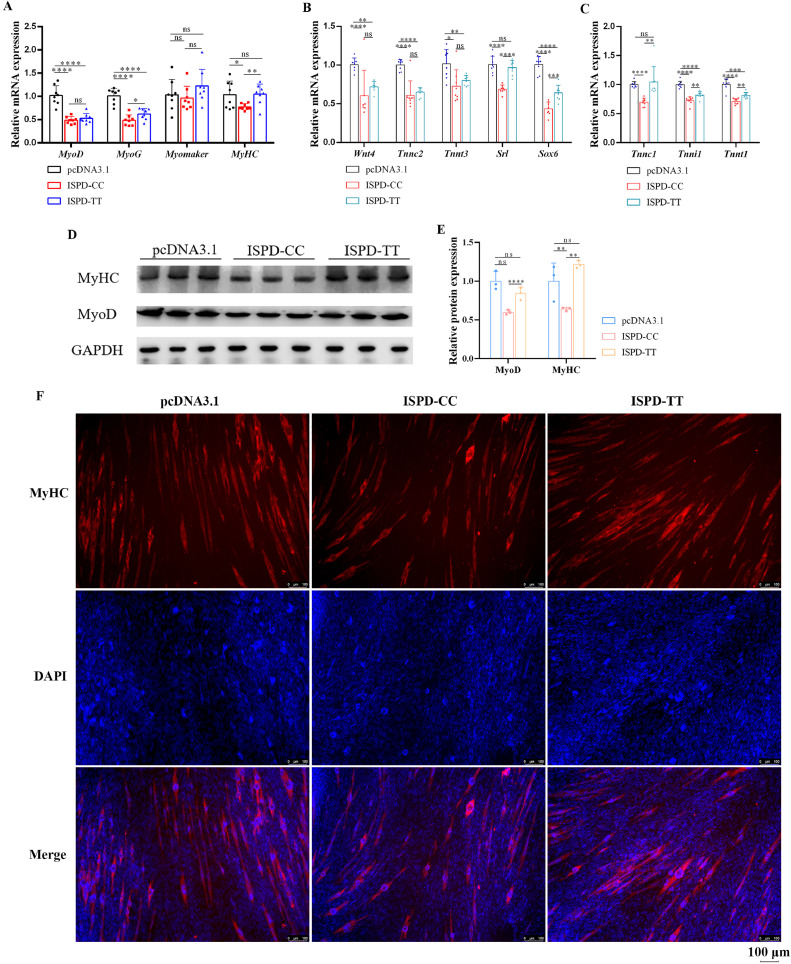
The missense mutation of Arg84Lys in *ISPD* diminishes myoblast differentiation inhibition. (A) The relative mRNA levels of myoblast differentiation-related genes. (B, C) The relative mRNA levels of fast growth muscle-related genes and slow growth muscle-related genes. (D) Protein expression of MyoD and MyHC. (E) Relative gray value of protein bands. (F) Immunofluorescence assay of MyHC.

Based on the association between *ISPD* alleles and skeletal muscle phenotypes, we speculated of a potential role on myoblast differentiation. The mRNA levels of myoblast differentiation marker genes (*MyoD, MyoG, Myomaker*, and *MyHC*) were quantified after ISPD-CC and ISPD-TT transfection and qRT-PCR result showed that the mRNA levels of *MyoD, MyoG, Myomaker* and *MyHC* were obviously downregulated by ISPD-CC, suggesting a negative effect of *ISPD* on myoblast differentiation (Figure 4A). However, ISPD-TT could release or reverse this downregulation, indicating that the missense mutation may affect skeletal muscle development through differentiation process regulation. In addition, we validated whether *ISPD* could influence myofibers types. The mRNA levels of myofibers transition related genes decreased, with disregard whether they are fast- or slow-muscles related genes (Figure 4B, C). A similar effect on MyoD and MyHC protein levels were also observed (Figure 3D, E). Compared with ISPD-CC, MyoD and MyHC protein levels were increased under ISPD-TT treatment. Considering the difference of myoblast differentiation inhibition between ISPD-CC and ISPD-TT, here we further explored whether these two types of *ISPD* have a difference on myotube formation. The immunofluorescence showed that ISPD-TT had a more obvious boost effect on the formation of myotubes, relative to ISPD-CC (Figure 4F). Collectively, the *ISPD* with “TT” allele genotype improves myoblast differentiation and myotube fusion.

### ISPD-TT Reduces Myotube Atrophy and Maintain Muscle Fibers Stability

**Figure 5:**
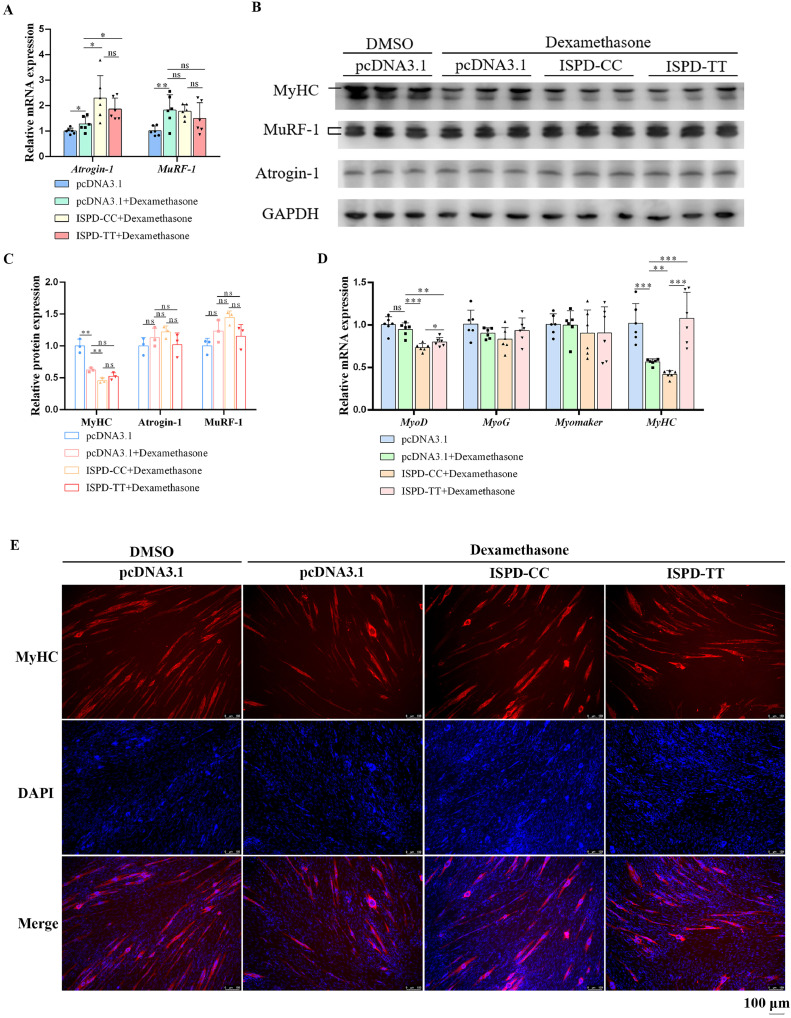
The missense mutation of Arg84Lys in *ISPD* maintains myofibers stability**.** (A) Relative mRNA levels of muscular atrophy-related genes. (B) Protein expression of MyHC, MurF-1 and Atrogin-1. (C) Relative gray value of MyHC, MurF-1 and Atrogin-1 protein bands. (D) Relative mRNA levels of myoblast differentiation-related genes. (E) Immunofluorescence assay of MyHC.

The muscle mass maintenance was controlled under the balance between protein synthesis and protein degradation. Thus, in addition to myogenesis, muscular atrophy, characterized by protein degradation, is another major way of controlling muscle mass. Here, dexamethasone was used to induce atrophy in myoblast. After dexamethasone treatment, the mRNA levels of muscle atrophy marker genes (*Atrogin-1* and *MuRF-1*) were significantly increased (Figure 5A). As expected, dexamethasone treatment also raised the protein levels of Atrogin-1 and MuRF-1 (Figure 5B, C), indicating that dexamethasone-mediated atrophy is occurring. To a certain extent, ISPD ectopic expression could increase Atrogin-1 and MuRF-1 expression under dexamethasone-mediated atrophy, manifesting *ISPD* positive effect on muscle atrophy. Compared with ISPD-CC, ISPD-TT showed a lower positive effect, again suggesting a promoting effect of missense mutation of Arg84Lys on reducing muscular atrophy. Subsequently, the myogenic supporting action on skeletal muscle was detected under muscle atrophy. With dexamethasone-mediated atrophy, only *MyHC* mRNA expression was obviously decreased (Figure 5D). At protein level, MyHC expression was also downregulated by dexamethasone (Figure 5B, C). Similarly, ISPD ectopic expression exacerbated MyHC expression reduction, while ISPD-TT could relieve this negative effect. Immunofluorescence results also provided an evidence that missense mutation of Arg84Lys could improve myoblast differentiation under muscular atrophy (Figure 5E). In total, missense mutation of Arg84Lys facilitated to maintain the muscle fibers stability.

## DISCUSSION

In this study, we preliminarily established a positive relationship between missense mutation of *ISPD* gene and muscle phenotype, analyzed the role of *ISPD* in myogenesis and muscular atrophy.

Glycosylation is an essential post-translational modification and the abnormal glycosylation on α-dystroglycan would cause muscular dystrophy (Kanagawa et al., 2016). Various missense mutations in *ISPD* were the main reason for muscular dystrophy due to its necessity on maintaining α-dystroglycan glycosylation (Cirak et al., 2013; Song et al., 2020; Bayram et al., 2021). Very interestingly, in our study, the missense mutation of Arg84Lys in *ISPD* was carried by Dongtao chicken, while the Bearded ones had 3 genotypes of this allele, suggesting a positive evolution in gamecock chicken. The results were in line with the data in our previous publication (Luo et al., 2020). And this missense mutation may have altered the structure of the protein in a way that led to changes in the muscle phenotype. Different to the cases in human limb-girdle muscular dystrophy, the missense mutation of Arg84Lys in chicken *ISPD* is beneficial to the muscle phenotype. This may due to the difference in the α-dystroglycan glycosylation between chicken and human. But it still needs further validation of the role of ISPD in chicken α-dystroglycan glycosylation process.

*ISPD* seems to be necessary to maintain muscle fiber homeostasis in mammals. Here, ISPD was confirmed to be a negative factor in myogenesis, while the mutated isoform could maintain myogenesis in contrast to ISPD-CC, suggesting that it is a favourable mutation for chicken muscle development. During myogenesis, myoblast proliferation and differentiation were generally an opposite physiological process (Guo et al., 2018; Luo et al., 2019). In this study, ISPD showed an unfavourable effect both in myoblast proliferation and differentiation. Our previous results have also shown that myoblast proliferation and differentiation are not necessarily opposite (Huang et al., 2019; Li et al., 2019). The missense mutation of Arg84Lys always alleviates or even reverses *ISPD* inhibition in myoblast proliferation and differentiation, which is consistent with the above association analysis between genotypes and muscle phenotypes. ISPD is widely known as Notch1-induced protein, indicating it may be an agonist for boosting Notch signal, while Notch signal suppresses MyoD stimulation on myoblast differentiation (Brohl et al., 2012), implying that ISPD may affect chicken myogenesis through regulating Notch signal. Compared with Arginine, Lysine was easier to be methylated on its residues. In addition, histones also exhibit acetylation diversity due to lysine, which is more conserved at the N-terminal. Therefore, the missense mutation may mediate greater Notch-induced activity due to lysine. Besides, compared with wild type ISPD, the missense mutation of Arg84Lys showed an effective decrease on MyHC degradation under muscular atrophy, again emphasizing an advantageous effect of this missense mutation on maintaining muscle fibers stability.

In summary, it was found that the missense mutation of Arg84Lys in *ISPD* was positively associated with chicken muscular phenotypes, and this mutation was able to alleviate myogenesis inhibition and maintain muscle fiber stability.

## Appendix Supplementary materials

Image, application 1
