# Supplementary material for: A missense mutation in ISPD contributes to maintain muscle fiber stability
Source: Poult Sci. 2022 Aug 31;101(11):102143. doi: 10.1016/j.psj.2022.102143 (PMC9513258; doi:10.1016/j.psj.2022.102143)
Supplement: Supplementary file 1 [file mmc1.docx]

Supplementary Table 1. Primers’ sequence information.

| Primers’ name | Sequence (5’-3’) | Product size (bp) | Annealing temperature (°C) |
| --- | --- | --- | --- |
| q-ISPD-F | AGAGGGTGAGCTGGATATCTGA | 230 | 60 |
| q-ISPD-R | TGGTCTCACAGCATCATGGA |  |  |
| q-Cyclin D1-F | CAGAAGTGCGAAGAGGAAGT | 188 | 58 |
| q-Cyclin D1-R | CTGATGGAGTTGTCGGTGTA |  |  |
| q-Cyclin D2-F | AACTTGCTCTACGACGACC | 150 | 58 |
| q-Cyclin D2-R | TTCACAGACCTCCAACATC |  |  |
| q-PCNA-F | GTGCTGGGACCTGGGTT | 217 | 58 |
| q-PCNA-R | CGTATCCGCATTGTCTTCT |  |  |
| q-Cyclin B2-F | CAGTAAAGGCTACGAAAG | 133 | 58 |
| q-Cyclin B2-R | ACATCCATAGGGACAGG |  |  |
| q-p27-F | GCTGTGCTGGGCTGAA | 207 | 58 |
| q-p27-R | GGACGAAAGGATGTGGG |  |  |
| q-p21-F | GAAGAGTTGTCCACGATAAGC | 247 | 58 |
| q-p21-R | TTCCAGTCCTCCTCAGTCC |  |  |
| q-GAPDH-F | CAACTTTGGCATTGTGGAGG | 130 | 55-62 |
| q-GAPDH-R | CGCTGGGATGATGTTCTGG |  |  |
| ISPD-F | TAAGAGCCTCGCAGAGTTGC | 1,661 | 58 |
| ISPD-R | TGAAGCTAGGAGTGAGGCTTG |  |  |

Supplementary Table 2. The association of rs313358571 with chicken growth phenotypes

| Traits | P-value | Phenotypes | | | |
| --- | --- | --- | --- | --- | --- |
|  |  | TT | TC | | CC |
| BW (g) | 0.9248 | 1642.56±13.98 (n=264) a | 1648.46±11.87 (n=344) b | 1692.7±20.47 (n=115) c | |
| SW (g) | 0.6041 | 1446.91±12.23 (n=263) a | 1451.66±10.42 (n=342) b | 1490.41±18.21 (n=114) c | |
| SR (%) | 0.1030 | 88.11±0.11 (n=263) | 88.17±0.10 (n=342) | 88.17±0.18 (n=114) | |
| HEW (g) | 0.4961 | 1302.4±11.71 (n=264) a | 1311.68±9.55(n=343) b | 1347.86±16.65 (n=115) c | |
| HEWR (%) | 0.7166 | 89.96±0.31 (n=263) | 90.37±0.09 (n=341) | 90.02±0.3 (n=114) | |
| EW (g) | 0.3050 | 1082.27±9.20 (n=264) a | 1084.68±7.95 (n=343) b | 1112.36±13.78 (n=115) c | |
| EWR (%) | 0.1951 | 74.74±0.14 (n=263) ab | 74.74±0.11 (n=341) a | 74.30±0.26 (n=114) b | |
| BMW (g) | 0.1419 | 84.24±0.8 (n=264) a | 84.03±0.66 (n=344) ab | 85.40±1.00 (n=115) b | |
| BMWR (%) | 0.3273 | 15.61±0.10 (n=264) | 15.54±0.08 (n=343) | 15.43±0.13 (n=115) | |
| LW (g) | 0.9017 | 193.72±1.91 (n=264) a | 195.23±1.65 (n=344) a | 200.92±2.79 (n=115) b | |
| LWR (%) | 0.2827 | 35.78±0.15 (n=264) | 35.91±0.11 (n=343) | 36.10±0.16 (n=115) | |
| BLR (%) | 0.6080 | 43.95±0.40 (n=264) | 44.28±0.91 (n=344) | 42.91±0.46 (n=115) | |
| WW (g) | 0.3789 | 64.38±0.56 (n=264) a | 64.21±0.47 (n=344) a | 65.28±0.95 (n=115) b | |
| WR (%) | 0.9258 | 11.91±0.04 (n=264) a | 11.85±0.03 (n=343) ab | 11.73±0.08 (n=115) b | |
| FW (g) | 0.3861 | 71.38±0.89 (n=264) a | 70.91±0.75 (n=344) a | 73.15±1.41 (n=115) b | |
| HW1 (g) | 0.7458 | 48.82±0.62 (n=264) a | 48.45±0.53 (n=344) a | 49.47±0.86 (n=115) b | |
| HW2 (g) | 0.1742 | 7.38±0.11 (n=263) a | 7.55±0.12 (n=344) ab | 7.63±0.18 (n=115) b | |
| LW2 (g) | 0.4215 | 32.37±0.37 (n=263) a | 32.89±0.35 (n=343) ab | 33.29±0.55 (n=115) b | |
| SW2 (g) | 0.6153 | 31.42±0.39 (n=264) | 30.98±0.32 (n=344) | 31.84±0.52 (n=115) | |
| AFW (g) | 0.0949 | 36.51±0.97 (n=264) a | 38.07±0.83 (n=344) a | 42.17±1.56 (n=115) b | |
| AFR (%) | 0.0958 | 2.23±0.06 (n=264) a | 2.31±0.05 (n=344) a | 2.50±0.09 (n=115) b | |
| SL (mm) | 0.2460 | 69.67±0.43 (n=264) a | 70.17±0.36 (n=343) b | 71.43±0.66 (n=114) c | |
| SC (mm) | 0.2423 | 11.27±0.07 (n=264) a | 11.11±0.06 (n=343) b | 11.20±0.10 (n=114) ab | |
| BL (cm) | 0.7314 | 19.48±0.07 (n=264) a | 19.49±0.06 (n=343) a | 19.71±0.11 (n=114) b | |
